# Early prognostic value of the lactate-to-albumin ratio in severe acute pancreatitis with acute respiratory distress syndrome

**DOI:** 10.3389/fmed.2026.1734599

**Published:** 2026-05-05

**Authors:** Qingcheng Zhu, Mingfeng Lu, Dingyu Tan, Min Xu

**Affiliations:** Department of Emergency Medicine, Northern Jiangsu People’s Hospital Affiliated to Yangzhou University, Yangzhou, China

**Keywords:** acute respiratory distress syndrome, lactate-to- albumin ratio, LAR, mortality, severe acute pancreatitis

## Abstract

**Background:**

Acute respiratory distress syndrome (ARDS) secondary to severe acute pancreatitis (SAP) is associated with high mortality; however, dependable biomarkers for early risk assessment remain scarce. The lactate-to-albumin ratio (LAR), reflecting both metabolic stress and nutritional status, may serve as a useful prognostic indicator in critical illness.

**Methods:**

This retrospective study included 351 SAP patients with ARDS who were admitted to the intensive care unit of a tertiary hospital from 2016 to 2024. Clinical and laboratory data within 24 h of admission were collected. The primary outcome was in-hospital mortality. A logistic regression analysis was performed to identify independent predictors, and receiver operating characteristic (ROC) curves were used to compare the prognostic performance of LAR, lactate, albumin, and Sequential Organ Failure Assessment (SOFA) scores.

**Results:**

In-hospital mortality was 23.6% (83/351). Non-survivors had significantly higher LAR levels than survivors (*p* < 0.001). After adjusting for confounders, LAR remained independently associated with mortality (OR = 1.32, 95% CI: 1.04–1.67, *p* = 0.024). ROC analysis showed that LAR outperformed albumin and achieved accuracy comparable to lactate and SOFA. High LAR (≥1.32) was associated with lower 28-day survival (*p* < 0.001). A threshold effect was identified at LAR = 1.78.

**Conclusion:**

LAR is an independent predictor of in-hospital mortality in SAP patients with ARDS. As a simple, rapid, and cost-effective biomarker, LAR may facilitate early risk stratification and clinical decision-making.

## 1 Introduction

Acute pancreatitis (AP) is an inflammatory disorder of the pancreas, with an annual incidence of approximately 30 cases per 100,000 men and 20 cases per 100,000 women in Western populations (1). Although a majority of the cases are mild and self-limiting, approximately 20% progress to severe acute pancreatitis (SAP), characterized by persistent organ failure and local complications such as pancreatic necrosis, abscesses, or pseudocyst formation (2). Despite substantial progress in critical care management, SAP continues to represent a major cause of morbidity and mortality in intensive care unit (ICU), with reported mortality rates ranging from 13 to 35% (3). Among its systemic complications, acute respiratory distress syndrome (ARDS) is particularly severe and is defined by hypoxemia and bilateral pulmonary infiltrates resulting from increased alveolar–capillary membrane permeability (4). ARDS develops in a considerable proportion of SAP patients and is associated with a mortality rate of approximately 30–40% (5), highlighting the urgent need for early risk stratification and reliable prognostic biomarkers in this population.

Several scoring systems, including the Acute Physiology and Chronic Health Evaluation II (APACHE II), the Ranson criteria, and the Sequential Organ Failure Assessment (SOFA), are commonly used to evaluate the severity of AP (6). Although these tools integrate multiple clinical and laboratory parameters to provide a comprehensive assessment, their complexity often requires extensive data collection, potentially delaying timely clinical decision-making (7). In emergency settings, such delays may impede early intervention and contribute to worse outcomes (8).

Lactate serves as an indicator of inadequate tissue perfusion and metabolic disturbance, and its prognostic significance has been widely explored in critically ill populations (9). In SAP, lactate clearance has been recognized as an independent determinant of mortality (10). Nonetheless, the interpretation of lactate levels may be influenced by confounding factors, such as sepsis, systemic inflammatory responses, and certain medications (11). Albumin, a negative acute-phase reactant, is also associated with disease severity, clinical outcomes, and risk of death (12). However, since albumin concentrations are affected by nutritional deficiencies and chronic inflammation, their value as a standalone prognostic marker is limited (13).

The lactate-to-albumin ratio (LAR) integrates lactate and albumin levels, offering a combined reflection of metabolic stress and nutritional condition (14). An increased LAR has been associated with unfavorable clinical outcomes across multiple critically ill cohorts, such as those with sepsis, traumatic brain injury, cardiac arrest, and acute respiratory failure (15, 16). Nevertheless, its prognostic significance in patients with SAP complicated by ARDS remains unclear. The present study aims to investigate the relationship between LAR and in-hospital mortality in this vulnerable population to improve early risk assessment and guide clinical management.

## 2 Materials and methods

### 2.1 Study design and eligibility

The study was carried out in a 78-bed ICU at a tertiary teaching hospital in Jiangsu Province, China. The research complied with the ethical guidelines of the Declaration of Helsinki and received approval from the Institutional Ethics Committee of Northern Jiangsu People’s Hospital (No. 20250328). Because of its retrospective nature, the ethics committee waived the requirement for written informed consent.

Patients with SAP complicated by ARDS between 1 January 2016 and 31 December 2024 were included in this study. AP was diagnosed according to the 2012 revised Atlanta criteria, which require at least two of the following: (1) typical abdominal pain, (2) serum amylase or lipase levels exceeding three times the upper normal limit, or (3) imaging findings indicative of AP (17). SAP was defined as organ failure persisting for more than 48 h, with a Marshall score of ≥ 2 in at least one organ system (respiratory, cardiovascular, or renal) (17). ARDS was identified based on the Berlin definition, requiring (1) acute onset (2), a PaO₂/FiO₂ ratio of ≤ 300 mmHg with a positive end-expiratory pressure ≥ 5 cm H₂O (3), bilateral pulmonary infiltrates on chest imaging, and (4) absence of cardiac failure. The severity of ARDS was categorized as mild (PaO₂/FiO₂ 201–300 mmHg), moderate (PaO₂/FiO₂ 101–200 mmHg), or severe (PaO₂/FiO₂ ≤ 100 mmHg) (18). ARDS was identified at ICU admission or within the first 72 h of the ICU stay to capture early-onset ARDS in patients with SAP. The diagnosis of ARDS was confirmed by experienced clinicians, based on clinical findings, arterial blood gas analysis, and chest imaging, according to the Berlin definition. All eligible patients were consecutively enrolled during the study period to reduce potential selection bias.

Adult patients (≥18 years) with complete clinical and biochemical data obtained within 24 h of hospital admission were enrolled. Exclusion criteria included pregnancy, presence of malignant tumors, previous pancreatic operations, or advanced liver cirrhosis with decompensation.

### 2.2 Data collection

Clinical information was obtained from electronic medical records, including demographic data, medical history, and admission vital signs—respiratory rate (RR), heart rate, systolic blood pressure (SBP), and diastolic blood pressure (DBP). The SOFA score at admission was also recorded. Laboratory results included hematologic parameters, liver and renal function tests, electrolytes, coagulation profiles [international normalized ratio, prothrombin time, activated partial thromboplastin time (APTT)], and arterial blood gas analyses (pH, partial pressures of oxygen and carbon dioxide, and lactate). Vasopressor use on admission was documented. The LAR was derived from corresponding laboratory measurements.

### 2.3 Primary outcome

The primary outcome was in-hospital mortality in SAP patients with ARDS. The secondary outcome was 28-day all-cause mortality, defined as death occurring within 28 days of ICU admission.

### 2.4 Statistical analysis

Continuous variables were first assessed for normality using the Kolmogorov–Smirnov test and summarized as mean ± standard deviation or median (interquartile range), depending on distribution. Group comparisons were conducted using Student’s t-test or Mann–Whitney U-test for continuous variables and the chi-square or Fisher’s exact test for categorical variables. Patients with incomplete key clinical or laboratory data within the first 24 h of admission were excluded from the analysis. Therefore, a complete case analysis approach was adopted.

Potential predictors of in-hospital mortality were initially screened using a univariable logistic regression analysis, with variables showing a *p*-value of < 0.05 subsequently entered into the multivariable logistic regression analysis to identify independent risk factors, reported as odds ratios (ORs) with 95% confidence intervals (CIs). Variable selection for multivariable models was based on a combination of univariable screening (*p* < 0.05), clinical relevance, and evidence from prior literature. Variables known to be associated with outcomes in SAP and ARDS were retained in the final models regardless of their univariable significance. Multicollinearity among variables was assessed using variance inflation factors (VIFs) in each multivariable model, and no significant collinearity was detected (all VIF values < 5). The discriminative performance of lactate, albumin, LAR, and SOFA scores was evaluated using receiver operating characteristic (ROC) curves, with the area under the curve (AUC), sensitivity, and specificity calculated for each parameter. Model calibration was assessed using the Hosmer–Lemeshow goodness-of-fit test for each multivariable model. The optimal LAR cutoff was determined using the Youden index, stratifying patients into high- and low-LAR groups, and survival differences were assessed through Kaplan–Meier analysis with the log-rank test. Subgroup analyses were conducted as exploratory and hypothesis-generating analyses and were not pre-specified in the study design. Therefore, the results should be interpreted with caution.

To explore potential non-linear relationships between LAR and mortality, smooth curve fitting and two-piecewise linear regression were performed, with inflection points identified via maximum likelihood estimation. All analyses were two-tailed, with a *p*-value of < 0.05 considered statistically significant, and were conducted using R (version 3.6.1) and SPSS (version 24.0).

## 3 Results

### 3.1 Baseline characteristics

**Figure 1:**
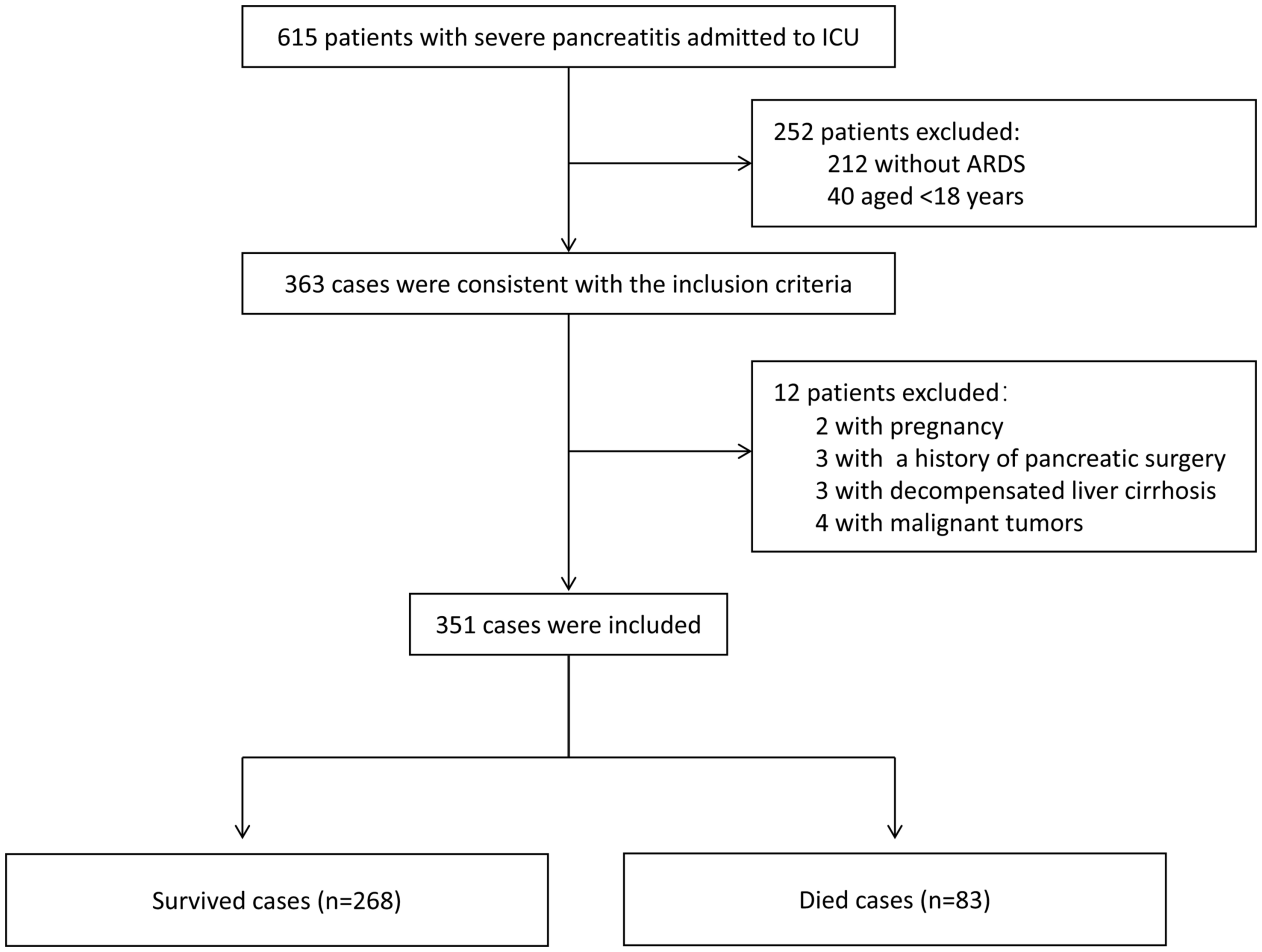
Flowchart of patient enrollment. ICU, intensive care unit; ARDS, acute respiratory distress syndrome.

During the study period, a total of 615 patients with SAP were screened. After applying the inclusion and exclusion criteria, 351 patients with SAP complicated by ARDS were included in the final analysis. The final cohort included 268 survivors and 83 non-survivors (Figure 1), with an overall in-hospital mortality of 23.6%. Baseline characteristics are shown in Supplementary Table S1.

Compared with survivors, non-survivors were significantly older (71 vs. 55 years, *p* < 0.001), and had lower SBP and DBP, but had higher RR and SOFA scores (all *p* < 0.05). Laboratory findings revealed markedly elevated LAR levels in non-survivors [1.34 (0.65–2.50) vs. 0.66 (0.43–1.17), *p* < 0.001], along with increased blood urea nitrogen, lactate, and APTT, and decreased arterial pH (all *p* < 0.05). The proportion of patients receiving vasopressors was also significantly higher among non-survivors (88.0% vs. 57.5%, *p* < 0.001). Other demographic, comorbidity, and biochemical parameters were comparable between the groups.

### 3.2 The LAR is an independent risk factor for hospital mortality

Variables with a *p*-value of < 0.05 in Supplementary Table S1 were entered in the univariate logistic regression analysis. As shown in Table 1, age, RR, SBP, DBP, SOFA score, blood urea nitrogen, APTT, arterial pH, lactate, LAR, and vasopressor use were all significantly associated with in-hospital mortality. Notably, unadjusted LAR demonstrated a strong association with mortality (OR, 1.64; 95% CI, 1.33–2.03; *p* < 0.001).

**Table 1:** Univariate logistic regression analysis of variables for hospital mortality.

Variables	Hospital mortality		
	OR	95% CI	*p*
Age	1.05	1.03–1.06	<0.001
Respiratory rate	1.04	1.01–1.07	0.013
Systolic blood pressure	0.98	0.97–0.99	0.003
Diastolic blood pressure	0.98	0.96–1.00	0.017
SOFA	1.16	1.08–1.23	<0.001
Blood urea nitrogen	1.01	1.00–1.02	0.018
Activated partial thromboplastin time	1.02	1.01–1.04	0.008
Arterial pH	0.10	0.02–0.61	0.013
Lactic acid	1.19	1.10–1.28	<0.001
LAR	1.64	1.33–2.03	<0.001
Vasopressor	5.40	2.67–10.92	<0.001

OR, Odds ratio; CI, Confidence interval; SOFA, Sequential Organ Failure Assessment; LAR, Lactate-to-albumin ratio.

In Model I, after adjusting for age, RR, SBP, and DBP, LAR remained an independent predictor of in-hospital mortality (OR, 1.57; 95% CI, 1.26–1.94; *p* < 0.001). This association persisted in fully adjusted Model II, which further controlled for SOFA score, blood urea nitrogen, APTT, arterial pH, and vasopressor use (OR, 1.32; 95% CI, 1.04–1.67; *p* = 0.024; Table 2). The Hosmer–Lemeshow test indicated good model fit for both multivariable models (Model I: *p* = 0.413; Model II: *p* = 0.368). No significant multicollinearity was observed among the variables included in the multivariable models (all VIF values < 5).

**Table 2:** Multivariate logistic analysis of risk factors for hospital mortality.

Variables	Non-adjusted		Adjust I		Adjust II	
	OR (95% CI)	*P*	OR (95% CI)	*p*	OR (95% CI)	*p*
LAR	1.64 (1.33–2.03)	<0.001	1.57 (1.26–1.94)	<0.001	1.32 (1.04–1.67)	0.024
LAR-group						
Low	Reference		Reference		Reference	
High	4.80 (2.83–8.17)	<0.001	4.80 (2.69–8.58)	<0.001	3.05 (1.52–6.12)	0.002

Non-adjusted model was adjusted for none; Adjust I model was adjusted for age, respiratory rate, systolic blood pressure, and diastolic blood pressure; Adjust II model was adjusted for age, respiratory rate, systolic blood pressure, diastolic blood pressure, SOFA, blood urea nitrogen, activated partial thromboplastin time, arterial pH, and the use of vasopressor. OR, Odds ratio; CI, Confidence interval; LAR, Lactate-to-albumin ratio.

LAR was analyzed both as a continuous variable and as a dichotomized variable based on the optimal cutoff value, with the dichotomized variable used for exploratory analysis to improve clinical applicability. To assess prognostic relevance, patients were stratified into low (<1.32) and high (≥1.32) LAR groups based on the optimal Youden index cutoff. Compared with the low-LAR group, high-LAR patients exhibited substantially higher in-hospital mortality across all models, with ORs of 4.80 (95% CI, 2.83–8.17; *p* < 0.001) in the unadjusted model, 4.80 (95% CI, 2.69–8.58; *p* < 0.001) in Model I, and 3.05 (95% CI, 1.52–6.12; *p* = 0.002) in Model II (Table 2).

### 3.3 ROC curve analysis

**Figure 2:**
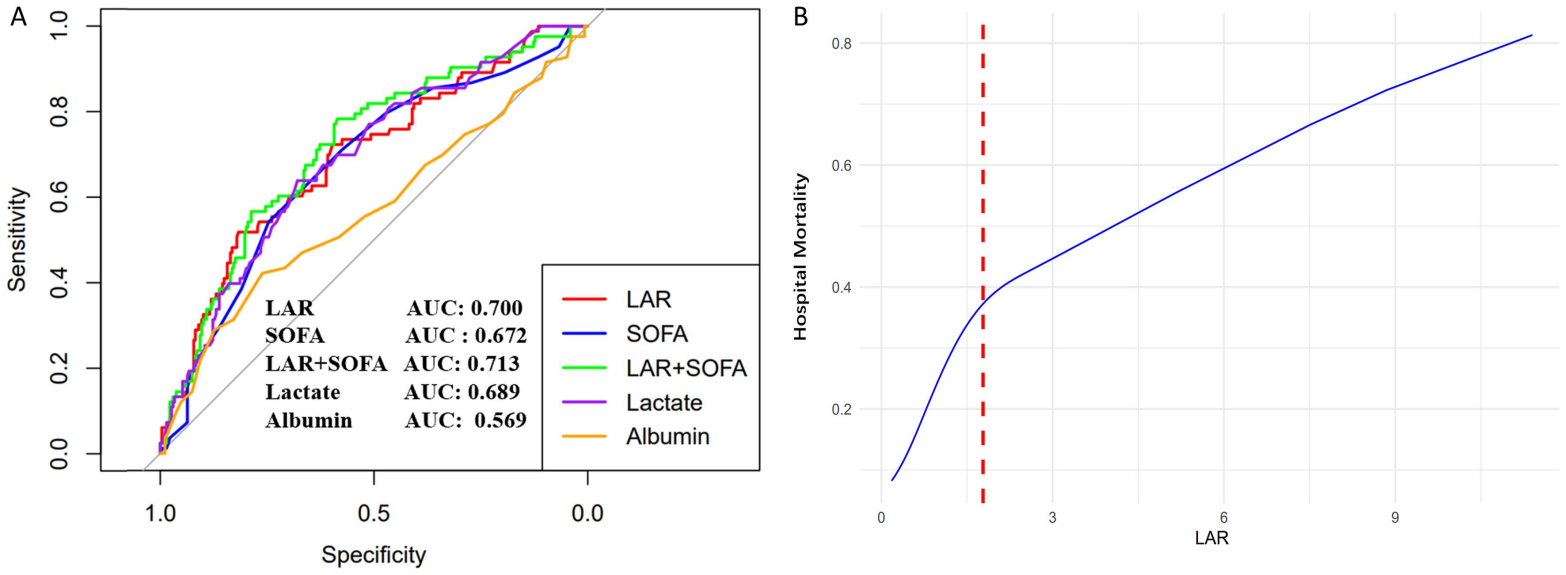
Association between LAR and hospital mortality in SAP patients with ARDS. **(A)** ROC curves assess the predictive capability of LAR for mortality. **(B)** The non-linear relationship between LAR and mortality. LAR, lactate-to-albumin ratio; SAP, severe acute pancreatitis; ARDS, acute respiratory distress syndrome; and ROC, receiver operating characteristic.

ROC curves were constructed to evaluate the predictive performance of LAR, lactate, albumin, SOFA score, and the combined LAR+SOFA model for in-hospital mortality in SAP patients with ARDS (Figure 2A). The AUCs were 0.700 (95% CI: 0.627–0.759) for LAR, 0.689 (95% CI: 0.625–0.753) for lactate, 0.569 (95% CI: 0.493–0.645) for albumin, and 0.672 (95% CI: 0.606–0.739) for SOFA. The combined LAR+SOFA model yielded the numerically highest AUC of 0.713 (95% CI: 0.650–0.777; Table 3). Pairwise comparisons showed that LAR significantly outperformed albumin (*p* < 0.001). However, no statistically significant differences were observed among LAR, SOFA, and the LAR+SOFA model (all *p* > 0.05).

**Table 3:** Information on ROC curves in Figure 2A.

Variables	Survived	Died	AUC (95% CI)	Specificity	Sensitivity	Youden’s index
LAR	268	83	0.700 (0.627–0.759)	0.817	0.518	0.335
Lactate	268	83	0.689 (0.625–0.753)	0.679	0.639	0.318
Albumin	268	83	0.569 (0.493–0.645)	0.951	0.072	0.023
SOFA	268	83	0.672 (0.606–0.739)	0.746	0.542	0.288
SOFA+LAR	268	83	0.713 (0.650–0.777)	0.586	0.783	0.369

ROC, Receiver operating characteristic; AUC, Area under the receiver operating characteristic curve; LAR, Lactate-to-albumin ratio; and SOFA, Sequential Organ Failure Assessment.

### 3.4 Kaplan–Meier curve analysis

**Figure 3:**
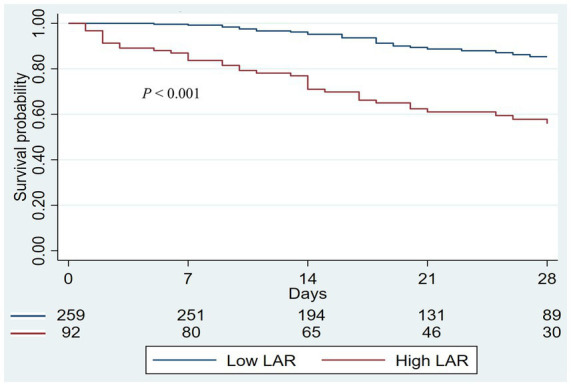
The Kaplan–Meier curves for 28-day survival in patients stratified into two groups according to the LAR: low (<1.32) and high (≥1.32). Numbers at risk at each time point are shown below the graph. LAR, lactate-to-albumin ratio.

Patients were stratified into low (<1.32) and high (≥1.32) LAR groups based on the optimal Youden index cutoff. The Kaplan–Meier analysis demonstrated a significantly lower 28-day survival in the high-LAR group than in the low-LAR group (*p* < 0.001; Figure 3).

### 3.5 The analysis of the non-linear association

A model adjusted for the covariates in Model II (Table 2) was constructed to examine the association between LAR and in-hospital mortality. After full adjustment, a non-linear relationship was observed (Figure 2B), with threshold effect analysis identifying a significant inflection point at 1.78. Below this threshold, LAR was strongly associated with mortality (OR, 2.63; 95% CI, 1.31–4.96; *p* = 0.007), whereas the association above the threshold was weaker but remained significant (OR, 1.21; 95% CI, 1.02–3.18; *p* = 0.015). The log-likelihood ratio test (*p* = 0.022) indicated that the two-piecewise linear regression model provided a superior fit compared with a single-line model (Table 4).

**Table 4:** Information on non-linear associations between LAR and in-hospital mortality in Figure 2B.

Outcome	Hospital mortality	
	OR (95% CI)	*p*
Model I: Fitting model by standard linear regression	1.64 (1.33–2.03)	<0.001
Model II: Fitting model by two-piecewise linear regression		
Inflection point	1.78	
≤ 1.78	2.63 (1.31–4.96)	0.007
> 1.78	1.21 (1.02–3.18)	0.015
*p* for log likelihood ratio test		0.022

LAR, Lactate-to-albumin ratio; OR, Odds ratio; and CI, Confidence interval.

The adjustment factors are in accordance with the adjust II model from Table 3.

### 3.6 LAR level and clinical outcomes

As shown in Table 5, patients in the high LAR group had significantly worse outcomes, including a markedly higher in-hospital mortality rate (46.7% vs. 15.4%, *p* < 0.001) and 28-day mortality rate (44.6% vs. 15.1%, *p* < 0.001), compared with those in the low LAR group. Moreover, the high LAR group exhibited longer ICU stays [15 (7–26) vs. 12 (6–21) days, *p* = 0.038] and prolonged hospital stays [21 (11–34) vs. 15 (11–26) days, *p* < 0.001]. These findings further demonstrate that elevated LAR is strongly associated with adverse clinical outcomes in SAP patients with ARDS.

**Table 5:** Relationship between LAR level and clinical outcomes.

Variables	LAR		*p*
	Low (<1.32)	High (≥1.32)	
*N*	259	92	
In-hospital mortality, *n* (%)	40 (15.4)	43 (46.7)	<0.001
28-day mortality, *n* (%)	39 (15.1)	41 (44.6)	<0.001
LOS ICU (days)	12 (6–21)	15 (7–26)	0.038
LOS hospital (days)	15 (11–26)	21 (11–34)	<0.001

LAR, Lactate-to-albumin ratio; LOS, Length of stay; ICU, Intensive care unit stay.

### 3.7 Subgroup analysis

**Figure 4:**
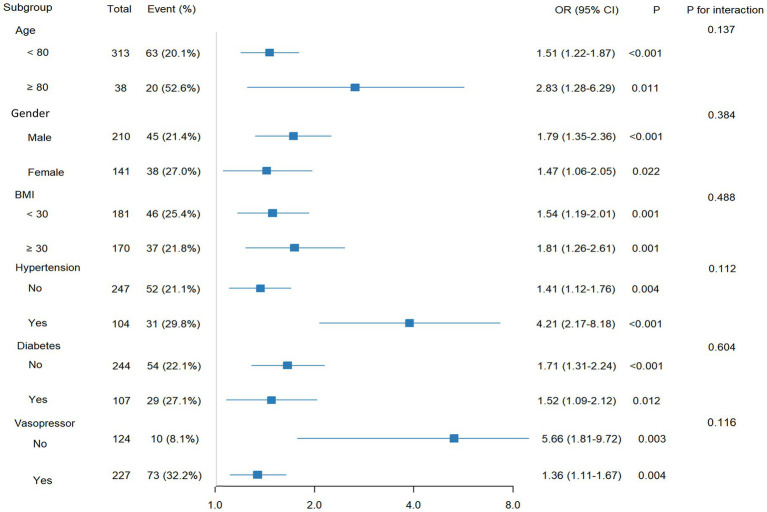
Forest plot for subgroup analysis of the relationship between hospital mortality and LAR. LAR, lactate-to-albumin ratio and BMI, body mass index.

To assess the robustness of the association between LAR and in-hospital mortality, subgroup analyses were conducted according to age, sex, body mass index (BMI), hypertension, diabetes mellitus, and vasopressor use (Figure 4). In all subgroups, elevated LAR was consistently associated with an increased risk of hospital mortality. No significant interactions were observed between LAR and any of these variables (all *p* for interaction > 0.05), suggesting that the association between high LAR and mortality was stable across different patient subgroups.

## 4 Discussion

ARDS represents a severe complication of SAP, markedly contributing to organ failure and increased mortality (19). To date, no study has specifically assessed the prognostic significance of the LAR in SAP patients with ARDS. In this retrospective cohort, LAR was identified as an independent predictor of in-hospital mortality. ROC analysis demonstrated that LAR (AUC = 0.700) performed better than albumin and slightly exceeded lactate and the SOFA score. The Kaplan–Meier analysis showed significantly reduced survival among patients with elevated LAR (≥1.32), while non-linear modeling revealed a distinct inflection point at 1.78. Collectively, these findings suggest that LAR serves as a simple yet reliable biomarker for predicting outcomes in SAP patients complicated by ARDS.

Clinical evidence indicates that nearly half of the patients with SAP develop ARDS, with an incidence of approximately 50–60% and a strong association to increased mortality (20). ARDS arises from a complex cascade initiated by AP, involving excessive inflammatory mediator release, cytokine storms, and disruption of the alveolar–capillary barrier, ultimately causing pulmonary edema, impaired gas exchange, and refractory hypoxemia (21). Among multiple organ failures associated with SAP, respiratory dysfunction is the most common and carries the highest mortality, accounting for the majority of early deaths (22). Experimental and clinical studies have further demonstrated that systemic inflammation, neutrophil activation, and endothelial injury contribute to the progression from SAP to ARDS, thereby exacerbating multi-organ dysfunction (23). Therefore, ARDS not only represents a marker of disease severity but also serves as an independent predictor of mortality, highlighting the urgent need for robust biomarkers to facilitate early risk assessment and guide timely clinical intervention in this vulnerable population.

Efforts to improve early risk assessment in AP have led to the development of multiple prognostic models. Conventional scoring systems—including the Ranson criteria, SOFA, and APACHE II—remain widely used to evaluate disease severity and guide treatment decisions (24). Despite their usefulness, these models provide only moderate predictive accuracy, with AUC values ranging generally between 0.6 and 0.8 for outcomes such as persistent organ failure or mortality (25). In addition, their reliance on parameters obtained within 48 h often limits the timely identification of high-risk patients (26). Consequently, researchers have turned to inflammation-related indicators, such as red cell distribution width, neutrophil-to-lymphocyte ratio, and C-reactive protein, as simpler alternatives (27). However, the prognostic relevance of these biomarkers remains uncertain, as findings across different studies are variable and often lack reproducibility, restricting their broader clinical application.

Lactate, generated during anaerobic metabolism, serves as an indicator of inadequate tissue oxygenation and perfusion (28). In the present cohort, elevated lactate was closely associated with increased hospital mortality among SAP patients complicated by ARDS, aligning with previous findings in critically ill populations. An analysis of the MIMIC-IV database by Zeng et al. identified lactate as an independent determinant of both short- and long-term mortality in AP (10). One study found that an arterial lactate concentration of ≥4 mmol/L was associated with a markedly increased risk of multi-organ failure, septic shock, and death (HR = 10, 95% CI: 3.7–27, *p* < 0.01) (29). However, lactate concentrations may not always reflect true tissue hypoperfusion, as they may also rise due to impaired hepatic metabolism or pharmacologic influences, such as *β*₂-agonists and metformin (30).

Albumin, the predominant plasma protein produced by the liver, is essential for maintaining physiological stability and oncotic balance (31). Low serum albumin has been consistently associated with higher in-hospital mortality in a large retrospective cohort of more than 20,000 emergency patients (32). Hypoalbuminemia is also frequently observed in AP, particularly in its severe forms, affecting nearly one-third of patients (33). Evidence from a study in Wuhan further showed that reduced albumin levels independently predicted persistent organ failure in AP (34). Unlike previous studies, our analysis did not identify hypoalbuminemia as an independent predictor of in-hospital mortality in patients with SAP complicated by ARDS. This discrepancy may be partly explained by the dynamic nature of albumin metabolism. Serum albumin levels can vary with nutritional status, fluid balance, and chronic inflammatory conditions (35), which may confound the association between serum albumin and adverse outcomes. Consequently, albumin alone may not accurately reflect the true disease severity in this population.

The LAR has emerged as a promising biomarker for outcome prediction in a range of critical conditions, including sepsis, heart failure, and respiratory failure, where elevated values are consistently linked to higher mortality (36). Previous research has shown that the LAR offers better prognostic accuracy for all-cause mortality than lactate or albumin alone in patients with AP (8). Similarly, in individuals with acute respiratory failure, LAR has been found to slightly outperform the SOFA score in predicting short-term mortality (37). Nevertheless, its prognostic performance in SAP complicated by ARDS remains unclear. In the present study, LAR exhibited stronger predictive capacity for in-hospital mortality than lactate, albumin, or SOFA, though the difference with SOFA was not statistically significant. Moreover, after complete adjusting for confounding variables, a non-linear association between LAR and mortality became evident. The non-linear association observed between LAR and in-hospital mortality indicates a potential threshold effect. From a clinical perspective, this threshold may serve as a practical cutoff to identify high-risk patients who may require intensified monitoring or early intervention. Nevertheless, this finding should be interpreted with caution and validated in future studies before clinical implementation. Collectively, these findings support the value of LAR as a potential tool for early risk assessment and stratification in SAP patients with ARDS.

This study has several limitations. First, as a single-center retrospective study, the generalizability of our findings may be limited, and causal relationships between LAR and in-hospital mortality in SAP patients with ARDS cannot be definitively established. Variations in ICU protocols, resuscitation strategies, and patient characteristics across institutions may further affect the applicability of our results. Second, although multiple confounders were adjusted for, potential bias from unmeasured factors may still exist. In particular, certain clinical factors, such as ARDS severity classification and detailed mechanical ventilation parameters, were not available in the dataset. Third, the relatively long study period may introduce potential bias due to temporal changes in ICU management, although treatment strategies in our center generally followed consistent guideline-based protocols. Fourth, only baseline LAR levels were analyzed, which might not fully capture the prognostic significance of dynamic changes in LAR during hospitalization. Fifth, subgroup analyses were exploratory in nature rather than pre-specified. Although these analyses were conducted to explore potential heterogeneity across clinically relevant subgroups, no formal adjustments for multiple comparisons were performed, which may increase the risk of type I error. Therefore, future multicenter prospective studies incorporating serial LAR measurements and a wider range of variables are warranted to further verify our results.

## 5 Conclusion

In SAP patients with ARDS, the LAR was independently associated with in-hospital mortality. LAR showed superior prognostic value over albumin and lactate, with accuracy comparable to SOFA. As an easily obtainable biomarker, it may assist in early risk stratification, although validation in prospective multicenter cohorts is warranted.

## Data Availability

The raw data supporting the conclusions of this article will be made available by the authors, without undue reservation.
