# Supplementary material for: Early prognostic value of the lactate-to-albumin ratio in severe acute pancreatitis with acute respiratory distress syndrome
Source: Front Med (Lausanne). 2026 May 5;13:1734599. doi: 10.3389/fmed.2026.1734599 (PMC13183856; doi:10.3389/fmed.2026.1734599)
Supplement: Supplementary file 1 [file Table_1.DOCX]

**Table S1** Baseline characteristics of selected patients

| Characteristics | Survived（n=268） | Died（n=83） | *P* |
| --- | --- | --- | --- |
| Female, n (%) | 103 (38.4) | 38 (45.8) | 0.287 |
| Age, years | 55(43-67) | 71(57-78) | <0.001 |
| Height (cm) | 170(163-178) | 168(160-177) | 0.156 |
| Weight (kg) | 85.2(73.0-100.0) | 84.0(72.1-98.3) | 0.238 |
| BMI (kg/m²) | 30(26-34) | 29(25-33) | 0.526 |
| Comorbidities, n (%) |  |  |  |
| COPD | 13 (4.9) | 4 (4.8) | 1.000 |
| Coronary artery disease | 42 (15.7) | 20 (24.1) | 0.111 |
| Hypertension | 73 (27.2) | 31 (37.3) | 0.104 |
| Diabetes mellitus | 78 (29.1) | 29 (34.9) | 0.383 |
| Vital signs |  |  |  |
| Respiratory rate (/min) | 24(18-30) | 27(21-34) | 0.004 |
| Heart rate (/min) | 109(90-127) | 114(96-132) | 0.237 |
| Systolic blood pressure (mmHg) | 105(94-121) | 100(79-114) | 0.002 |
| Diastolic blood pressure (mmHg) | 56(49-65) | 54(40-60) | 0.011 |
| SOFA | 8(5-11) | 11(8-13) | <0.001 |
| Berlin classification, n (%) |  |  | 0.240 |
| Mild | 136(50.7) | 39(47.0) |  |
| Moderate | 81(30.2) | 36(43.4) |  |
| Severe | 42(15.7) | 17(20.5) |  |
| Laboratory data |  |  |  |
| Red blood cell (*10^12^/L) | 3.8(3.3-4.3) | 3.7(3.1-4.4) | 0.422 |
| White blood cell (*10^9^/L) | 12.8(8.6-18.1) | 12.6(7.1-17.8) | 0.570 |
| Platelet (*10^9^/L) | 193(140-258) | 198(114-287) | 0.595 |
| Hemoglobin (g/L) | 11.7(10.2-13.5) | 11.1(9.7-13.0) | 0.102 |
| Total bilirubin (mg/dL) | 1.0(0.5-1.9) | 1.1(0.7-3.3) | 0.101 |
| Alanine aminotransferase (U/L) | 43(24-136) | 44 (26-119) | 0.969 |
| Aspartate aminotransferase (U/L) | 62(34-179) | 79 (36-216) | 0.299 |
| Lactate dehydrogenase (U/L) | 397 (276-610) | 397 (278-656) | 0.589 |
| Creatinine (mg/dL) | 1.1(0.8-2.1) | 1.3(0.9-2.2) | 0.160 |
| Blood urea nitrogen (mmol/L) | 21(13-36) | 29(19-48) | 0.003 |
| Albumin (g/dL) | 2.9(2.5-3.3) | 2.7(2.2-3.3) | 0.057 |
| Sodium (mmol/L) | 138(135-142) | 138(134-141) | 0.228 |
| Potassium (mmol/L) | 4.1(3.7-4.6) | 4.2(3.8-5.1) | 0.327 |
| Chloride(mmol/L) | 104 (99-110) | 105 (100-109) | 0.865 |
| Calcium (mmol/L) | 1.98(1.79-2.15) | 2.00(1.83-2.13) | 0.620 |
| Glucose (mmol/L) | 7.3(5. 8-10.2) | 7.3(6.1-9.7) | 0.630 |
| Amylase (U/L) | 156(80-250) | 156(66-287) | 0.557 |
| Lipase (U/L) | 184(50-613) | 191(50-904) | 0.475 |
| International normalized ratio | 1.3(1.1-1.5) | 1.3(1.1-1.7) | 0.256 |
| Prothrombin time (seconds) | 14.4(13.0-16.4) | 14.8(13.4-18.4) | 0.242 |
| Activated partial thromboplastin time (seconds) | 30.3(26.9-36.3) | 32.5(28.4-42.4) | 0.011 |
| Arterial pH | 7.3(7.2-7.4) | 7.3(7.2-7.4) | 0.019 |
| PaO_2_ (mmHg) | 80(68-106) | 81(68-104) | 0.840 |
| PaCO_2_ (mmHg) | 43(36-50) | 42(34-52) | 0.576 |
| Lactate (mmol/L) | 1.8(1.3-3.3) | 3.4(2.0-5.6) | <0.001 |
| LAR | 0.66(0.43-1.17) | 1.34(0.65-2.50) | <0.001 |
| Vasopressor, n (%) | 154 (57.5) | 73 (88.0) | <0.001 |

Abbreviations: BMI, Body mass index; COPD, Chronic obstructive pulmonary disease; SOFA, Sequential Organ Failure Assessment; PaO_2,_ Partial pressure of arterial oxygen; PaCO_2_, Partial pressure of arterial carbon dioxide; LAR, Lactate-to-albumin ratio.
